# Phase-to-Phase With Nucleoli – Stress Responses, Protein Aggregation and Novel Roles of RNA

**DOI:** 10.3389/fncel.2019.00151

**Published:** 2019-04-26

**Authors:** Leena Latonen

**Affiliations:** Institute of Biomedicine, University of Eastern Finland, Kuopio, Finland

**Keywords:** nucleoli, stress responses, protein aggregation, amyloidosis, proteasome inhibition, non-coding RNA

## Abstract

Protein- and RNA-containing foci and aggregates are a hallmark of many age- and mutation-related neurodegenerative diseases. This article focuses on the role the nucleolus has as a hub in macromolecule regulation in the mammalian nucleus. The nucleolus has a well-established role in ribosome biogenesis and functions in several types of cellular stress responses. In addition to known reactions to DNA damaging and transcription inhibiting stresses, there is an emerging role of the nucleolus especially in responses to proteotoxic stress such as heat shock and inhibition of proteasome function. The nucleolus serves as an active regulatory site for detention of extranucleolar proteins. This takes place in nucleolar cavities and manifests in protein and RNA collections referred to as intranucleolar bodies (INBs), nucleolar aggresomes or amyloid bodies (A-bodies), depending on stress type, severity of accumulation, and material propensities of the macromolecular collections. These indicate a relevance of nucleolar function and regulation in neurodegeneration-related cellular events, but also provide surprising connections with cancer-related pathways. Yet, the molecular mechanisms governing these processes remain largely undefined. In this article, the nucleolus as the site of protein and RNA accumulation and as a possible protective organelle for nuclear proteins during stress is viewed. In addition, recent evidence of liquid-liquid phase separation (LLPS) and liquid-solid phase transition in the formation of nucleoli and its stress responses, respectively, are discussed, along with the increasingly indicated role and open questions for noncoding RNA species in these events.

## Introduction

**FIGURE 1:**
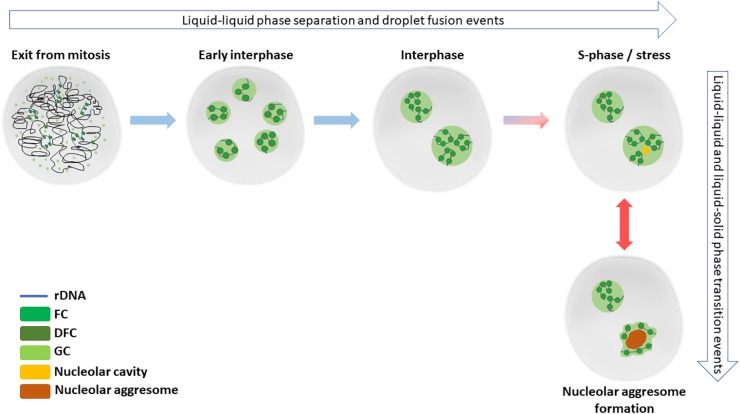
Nucleolar formation and stress responses involve phase separation and transition events. After cell division in late mitosis, nucleoli start to reform by reactivated rRNA transcription and liquid-liquid phase separation events. By early interphase, each individual NOR containing the rDNA repeats are surrounded by a functional nucleolus. Later during the interphase, the small nucleoli fuse to typically form 1–2 mature nucleoli in diploid, non-transformed cells. Nucleolar cavities can be detected in S-phase cells or upon cellular stress, such as DNA damage. When cells are exposed to severe proteotoxic or e.g., heat stress, nucleolar aggressomes and amyloid bodies are formed within one or more nucleoli of a nucleus, involving liquid-solid transition of aggregate contents.

Nucleoli are the site of ribosome biogenesis. They are formed in nuclei around tandem head-to-tail gene repeats of ribosomal DNA (rDNA) in the so called nucleolar organizing regions (NORs). In human cells, NORs are located in the short arms of five acrocentric chromosomes, and their size ranges from 50 kb to >6 Mb (Mangan et al., 2017). The nucleoli are initiated upon and structurally depend on active transcription of rDNA. The nucleoli are dispersed during mitosis as the rDNA transcription is halted. During telophase, the rDNA transcription resumes, and the nucleoli begin to reform as small nucleoli around individual NORs. As the cell cycle progresses, nucleoli fuse, forming larger, mature nucleoli containing multiple NORs (Hernandez-Verdun, 2011; Figure 1).

In human cells, the mature nucleoli are associated with perinucleolar heterochromatin (PNH), DNA sequences located distal and proximal to NORs on the acrocentric chromosomal arms (McStay, 2016), which is likely to contribute to positioning of the nucleoli to the 3D context in the nuclei. Currently, the sequences of the acrocentric arms are missing from human genome drafts. Yet, what is known is that the sequences on the centromeric side of rDNA are heavily segmentally duplicated and likely do not contain NOR regulatory elements (Floutsakou et al., 2013; Mangan et al., 2017). The telomeric sides of NORs contain regions called distal junctions (DJs). Their sequences are shared between the acrocentric chromosomes and dominated by around 100 kb inverted repeats and seem to have a complex chromatin structure (Floutsakou et al., 2013; Mangan et al., 2017). DJ sequences have been suggested to anchor rDNA to the PNH (Mangan et al., 2017). Other anchors for the spatial positioning of the nucleoli are intermediate filament proteins, especially lamins A/C, B1 and B2, that connect the nucleoli to nuclear matrix and contribute to maintaining nucleolar structure and functions (Martin et al., 2009; Louvet et al., 2014; Matsumoto et al., 2016; Buchwalter and Hetzer, 2017; Sen Gupta and Sengupta, 2017).

The nucleoli belong to a group of membraneless organelles (MLOs), and as such, they are dynamic structures with highly mobile constituents that can diffuse in and out to the nucleoplasm. Recently, the role of liquid-liquid phase separation (LLPS) in formation of MLOs has been increasingly recognized (Shin and Brangwynne, 2017; Sawyer et al., 2018). LLPS has a role in the formation and internal organization of the nucleoli to functional substructures (Feric et al., 2016). The current view thus holds that formation of the nucleoli is a combination of both active recruitment of factors and LLPS.

The nucleoli have a tripartite structure (Figure 1) with the three substructures functionally separate. The fibrillar centers (FCs) contain non-transcribed rDNA and rDNA chromatin associated factors. The rDNA transcription occurs at the interface between FCs and dense fibrillary component (DFC), in the latter of which occurs the early processing of precursor ribosomal RNA (rRNA). Late processing of rRNA and assembly of ribosome units takes place at the granular component (GC), surrounding the FCs and DFCs. Interestingly, yeast and other lower eukaryotes lack FCs, which may be connected to the closed nuclear division and intactness of the nucleoli through the cell cycle (Thiry and Lafontaine, 2005).

The tripartite nucleolar structure in human cells depends on the active transcription of rDNA, as several studies have shown inhibition of rDNA transcription by RNA polymerase I (RNApolI) disperses the nucleoli (reviewed in Grummt, 2013). The start of rRNA transcription has long been thought to be the initiating event for nucleolar reformation at the end of mitosis. This view was challenged by Dousset et al. (2000), who’s work indicate that postmitotic nucleologenesis results from direct recruitment of processing factors and pre-rRNAs to UBF-associated NORs before or at the onset of rDNA transcription. This is followed by fusion of prepackaged prenucleolar bodies into the nucleolus, suggesting that pre-ribosomal ribonucleoproteins synthesized in the previous cell cycle may contribute to nucleolar formation at the end of mitosis (Dousset et al., 2000).

## Nucleolar Contents and LLPS

The nucleolus is packed with protein – protein density in nucleoli is approximately double of that of the nucleoplasm (Handwerger and Gall, 2006). Although very dense, the nucleolus is also very dynamic: many nucleolar proteins are constantly moving between the nucleolus and the nucleoplasm (Leung and Lamond, 2003; Hernandez-Verdun, 2006; Sirri et al., 2008). The proteome of the nucleolus before and after stress is well described (Andersen et al., 2005; Moore et al., 2011). Most nucleolar molecules function in transcription and different maturation steps of rRNA (Andersen et al., 2005). However, there are at least dozens, if not hundreds, of nucleolar proteins with no apparent role in the formation of ribosomes (Andersen et al., 2005). Recently, it has become clear that the nucleolus contributes to biogenesis of multiple ribonucleoprotein particles, and the regulation of cellular events such as mitosis, the cell-cycle, and responses to several types of stress (Boisvert et al., 2007; Boulon et al., 2010; Lindström and Latonen, 2013).

RNA content of the nucleoli is not fully described. The well-recognized components, such as rRNA and snoRNA, are well known for their functions in ribosome production, but other non-coding components are not comprehensively described. In addition to the traditionally viewed roles in processing pre-rRNA and formation of ribosomal particles, nucleolar RNA is increasingly seen to have a role through contributing to nucleolar formation through promoting LLPS (Sawyer et al., 2018). MLOs typically harbor specific RNAs and intrinsically disordered, multivalent hub proteins, both contributing to the LLPS characteristics (Sawyer et al., 2018). It has been shown that the disordered domains in FBL and NPM (key components of DFC and GC, respectively) are required for droplet formation, and that RNA recognition motifs are required for maintaining phase separation (Brangwynne et al., 2011; Mangan et al., 2017). The sequence-encoded features of these proteins influencing their LLPS behavior also lie behind nucleolar compartmentalization, driven by different biophysical properties of the droplets, especially surface tensions (Feric et al., 2016). While specific RNAs themselves may be capable of phase separation as in the case of e.g., extended repetitive RNA motifs in clinical disorders, LLPS for MLOs is viewed to be driven more by RNA-protein interactions than RNAs as such (Sawyer et al., 2018). Long RNA molecules may potentially interact with several other proteins and RNAs simultaneously, favoring and strengthening the interactions between droplet-forming proteins. In addition, RNA-protein ratio and RNA multivalency may also be critical factors for MLO LLPS (reviewed in Sawyer et al., 2018). Interactions between NPM and rRNA promote LLPS in the nucleolar formation and supports the idea of active rDNA transcription spatially and temporally coordinating with critical, intrinsically disordered region (IDR)-containing LLPS drivers (Mitrea et al., 2018; Sawyer et al., 2018). It is likely that, in addition to rRNA, there are other contributing RNA species for LLPS in nucleolar formation yet to be identified. Likely candidates are at least the lncRNAs coded by the DJ regions (Mangan et al., 2017).

Changes in relative levels of the RNA components, likely to have profounding effects of nucleolar activity as well as organization in terms of LLPS, are not well known. Nucleolar rRNA is so abundant compared to other RNA species in the cell in general that rRNA sequences are often excluded in sequencing assays. The sequence repetitivity and lack of the reference genomes for rDNA areas makes it currently infeasible to align rRNA sequences for most quantitative expression analyses via next generation sequencing approaches. Most importantly, the lack of NORs and adjacent regions from genomic assemblies hampers the expression analyses of these areas and studies for the roles of the ncRNA expressed from these.

## Nucleolar Alterations Upon DNA Damage and Transcriptional Stress

**FIGURE 2:**
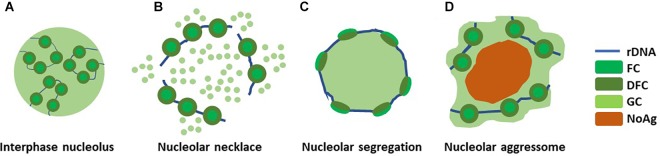
Nucleolar reorganization upon stress. The nucleolus reacts to different types of stress by structural deformations. **(A)** A normal interphase nucleolus under homeostasis shows tripartite structure composed around rDNA. **(B)** Upon DRB treatment, when RNApolI transcription remains active but rRNA processing is impaired, so called nucleolar necklaces are formed. **(C)** Nucleolar segregation, or nucleolar caps, are formed when RNApolI transcription is inactivated, e.g., with Actinomycin D. **(D)** Nucleolar aggresomes are formed within the nucleolus, in the nucleolar detention centers, upon proteotoxic insults such as proteasome inhibition and heat schock. This may or may not involve inhibited RNApolI activity.

Nucleolar structure changes significantly in response to several types of stress (Figure 2). If and when the rRNA production is halted resulting from e.g., double strand break-inducing DNA damage by ionizing radiation (IR) or RNA pol I inhibition by actinomycin D, nucleolar segregation occurs and so called nucleolar caps are formed. Nucleolar caps are bipartite structures containing FCs and DFCs which surround the GC components (Puvion-Dutilleul et al., 1992). A different structural reorganization, the nucleolar necklace, is formed under certain conditions where RNApolI transcription remains active, but rRNA processing is impaired (Figure 2). This is evident upon treatment of cells with doxorubicin (DRB), a DNA intercalating agent and inhibitor of DNA topoisomerase II (Louvet et al., 2005).

DNA damage in the form DNA bulges, by e.g., various cytotoxic drugs or UV radiation, cause inhibition of rDNA transcription by RNApolI, resulting in nucleolar disruption. The dispersal of the nucleolus releases proteins to nucleoplasm that normally do not reside in there, a mechanism by which certain stress responses are induced. E.g., p53 nucleolar and ribosomal proteins binds to the MDM2 protein following disruption of ribosome biogenesis. This leads to inhibition of MDM2 E3 ligase activity and thus to p53 activation (reviewed in Lindström and Latonen, 2013). It is interesting that the nucleolar responses to UV and IR differ (Moore et al., 2011). In addition to the different DNA damage types, these insults induce also damage to other macromolecules and partly different cellular responses (Laiho and Latonen, 2003; Goldstein and Kastan, 2015). Currently, it is unclear which other cellular events are involved in dictating the differential nucleolar stress responses upon these stresses (Moore et al., 2011).

It is well established that disruption of the nucleolus triggers a p53-dependent cellular stress response referred to as “nucleolar stress” (Zhang and Lu, 2009; Lindström and Latonen, 2013). This is frequently called also “ribosomal stress,” although not all abnormalities in ribosome biogenesis lead to dispersal of the nucleolus. Nucleolar and/or ribosomal stress, mediated to a large extent by interactions of translocated ribosomal and other nucleolar proteins and rRNA, activates signaling pathways leading to cell cycle arrest, apoptosis, differentiation or senescence, in a cell type and stress severity-dependent manner (reviewed in Lindström and Latonen, 2013).

Translocation to the nucleolus is also a regulatory mechanism under several cellular conditions. Initially, nucleolar sequestration as a concept was introduced by Bachant and Elledge (1999) based on work showing that exit from mitosis in budding yeast is regulated by detention of Cdc14 in the nucleolus (Shou et al., 1999; Visintin et al., 1999). The concept was further supported by the notion that, in mammalian cells, tumor suppressor Arf sequesters Mdm2 in the nucleolus to ensure activation of p53 during oncogene activation and replicative senescence (Weber et al., 1999). Detention in the nucleolus has been described for many proteins especially under different stress conditions. For example, MDM2, which is a ubiquitin ligase for tumor suppressor p53 among others, localizes to the nucleolus also upon transcriptional inhibition by Actinomycin D and possesses lower mobility there (Lohrum et al., 2003; Kurki et al., 2004; Mekhail et al., 2005). MDM2 is also transferred to nucleoli upon DNA damage by PML in an ARF-dependent manner (Bernardi et al., 2004). DNA damage induces translocation of also other proteins to nucleoli. For example, IR restores the disturbed association of telomerase protein with the nucleoli in transformed cells (Wong et al., 2002). Acidosis triggers pH-dependent interaction von Hippel-Lindau tumor suppressor protein (VHL) with rDNA, a phenomenon which is promoted by activation of hypoxia inducible factor HIF (Mekhail et al., 2004a,b, 2006). The authors suggest that this is a way for oxygen-starved cells to maintain energy equilibrium by gauging the environmental H+ concentration to statically retain VHL in nucleoli to restrict ribosomal production (Mekhail et al., 2006).

## Nucleolar Detention Under Protein Stress – Aggresomes, Amyloidogenesis and Relevance to Disease

Different from insults directly affecting rRNA transcription and processing, protein stress causes extranucleolar proteins and RNA to be detained in the nucleolus. Initially, certain stress-responsive proteins, such as p53, Mdm2 and PML body proteins, were described to translocate to nucleoli stress-signal-dependently (Klibanov et al., 2001; Mattsson et al., 2001; Xirodimas et al., 2001; Latonen et al., 2003). Later, this phenomenon was described to apply to a number of nuclear UPS client proteins and represent formation a *de novo* stress response organelle (Latonen, 2011; Latonen et al., 2011).

This foci formation takes place in nucleolar cavities, and intranucleolar bodies (INBs) can already be detected in S-phase cells and even after certain types of DNA damage (Abella et al., 2010; Hutten et al., 2011). Upon severe protein stress upon e.g., heat shock, chemical inhibition of proteasome activity, and acidosis, an expanded organelle is formed (Latonen et al., 2011; Audas et al., 2012a, 2016). The intranucleolar stress-responsive macromolecular collections have also been called to occur in so called detention centers, and intranucleolar macromolecular collections showing amyloid properties have been termed amyloid bodies (A-bodies) (Jacob et al., 2013; Audas et al., 2016).

Currently it is not clear how these structures relate to each other, but they share striking similarities: (1) they all form in the nucleoli but are clearly not belonging to normal components of the nucleoli, (2) they involve accumulation of protein which are not normal components of the traditional nucleolar structures, and many of these deposits have been shown to contain at least some of the same proteins, (3) often there is also RNA, which does not belong to normal nucleolar components, accumulating, and (4) formation of many of these structures has been shown to depend on intactness of the nucleoli, with the help experiments utilizing Actinomycin D-mediated nucleolar disruption. Thus, although INBs, nucleolar aggresomes and A-bodies have not been proven to represent same structures, with such striking similarities it seems plausible that they represent a range of sizes and states resulting from same phenomena.

The initial papers describe events occurring upon different cellular stresses (proteasome inhibition, acidosis, heat stress, DNA damage), and certain differences exist in the contents of the organelles. Thus, cellular context- and stress-dependency of RNA and protein recruitment remains to be investigated in future studies. The common denominator seems to be to clear nuclear proteins from the nucleoplasm to regulate cellular activities for the duration of the stress situation, and at least upon certain insults, the formation of the intranucleolar collections can be transient. For certain proteins, there is evidence for functional impact in the localization to nucleolar aggressomes, such as for TTRAP, which regulates rRNA processing during cellular response to proteasome inhibition (Vilotti et al., 2012). Considering, however, that nucleolar aggresomes can form even as a result of overexpression of exogenous proteins or increased protein synthesis due to a viral infection (reviewed in Latonen, 2011), the role of the nucleolar aggresomes may be, at least at times, to protect the nucleoplasmic environment from excess proteins. In fact, the formation of A-bodies has been suggested as a form of so called protective or functional amyloidosis (Lyons and Anderson, 2016; Woodruff et al., 2018). Amyloid-bodies are solid condensates (Woodruff et al., 2018), and as such, resemble Balbiani-bodies in Xenopus oocytes, forming by amyloid-like assembly of a disordered protein Xvelo (Boke et al., 2016). Proteins in nucleolar aggresomes exhibit decreased mobility (Latonen et al., 2011), while INBs are likely soluble, exhibiting liquid-like spherical appearance (Hutten et al., 2011).

Thus, a plausible, yet speculative, sequence of events (Figure 2) in nucleolar aggresome formation involves an initial liquid phase in the nucleolar cavity or detention center (Wang M. et al., 2018). With prolonged accumulation of macromolecules to the structure, the proteins turn immobile (Latonen et al., 2011), liquid-solid phase transition occurs, and may proceed to amyloidogenesis (Audas et al., 2016). RNA seeding is involved in the seeding, at least for the amyloidogenic phase (Audas et al., 2016; Lyons and Anderson, 2016). It is possible that amorphous gel like intermediate states also exist to maturate concentrates of initially liquid state (Woodruff et al., 2018), although this is currently purely speculative. Thus, the exact material properties in each condition, and the mechanisms leading to the possible phase transitions remain to be investigated.

It seems that the composition of the nucleolar aggresomes is somewhat dependent on the stress insult. In general, the proteins are collected to the nucleolar aggresome along with RNA, and most often the aggresomes contain conjugated ubiquitin, SUMO, and heat shock factors (reviewed in Latonen, 2011). Although nucleolar aggresomes bare similarities with cytoplasmic aggresomes especially in the presence of ubiquitin conjugates, heat shock factors and links to hampered protein degradation, they are clearly different structures from cytoplasmic aggresomes (Latonen, 2011). Furthermore, inhibition of lysosomal proteases does not affect nucleolar aggresomes (Latonen et al., 2011; Salmina et al., 2017), indicating that nucleolar aggresome formation is separate from general protein degradation defects. Nucleolar aggresomes can, however, be released to the cytoplasm during mitosis and processed through the autophagocytosis pathway (Salmina et al., 2017). Nucleolar aggresomes can occur in several cell types, their prominence being greatest in normal diploid cells (Latonen et al., 2011). It is possible that the proliferative activity and transformation status of the cells affecting nucleolar activity and organization also affects formation of nucleolar aggresomes.

The roles and identities of the RNA components in nucleolar aggresomes remain to be investigated fully. Non-coding RNA transcribed from rDNA (IGS_16_RNA, IGS_22_RNA and IGS_28_RNA) has been shown to recruit proteins to aggresomes upon hypoxia/acidosis and heat shock (Audas et al., 2012a; Jacob et al., 2012, 2013). Nucleolar aggresomes formed after proteasome inhibition contain polyA-tailed RNA, suggestive of either mRNA, lncRNA or both (Latonen et al., 2011). Due to lack of comprehensive extraction and sequencing studies, the full range of RNA species localized in nucleolar aggresomes is yet to be discovered.

While nucleolar aggresomes formed upon proteasome inhibition or protein overexpression have not yet been shown to be reversible, this has been reported for acidosis and heat shock-induced events (Audas et al., 2016). In addition, certain nucleolar aggresome-inducing stress events seem to inhibit rDNA transcription (Jacob et al., 2013) while others do not (Latonen et al., 2011). Thus, inhibition of rDNA transcription may not be necessary for nucleolar aggresomes to form, but a co-occuring or a following event under certain stress conditions.

### Nucleolar Aggresomes and Neurodegeneration

The formation of nucleolar aggresomes in cultured cells resembles – and models – the situation in certain neurodegenerative disorders where proteins and RNA accumulate and aggregate to nuclei of cells, such as Huntington’s disease (HD) and spinocerebellar ataxias (SCA). A hallmark of numerous neurodegenerative diseases is ubiquitin, SUMO- and RNA-containing inclusion bodies that link to expression of aggregation-prone mutant forms of disease-related proteins or RNA and to impairment of UPS (Dorval and Fraser, 2007; Lehman, 2009; Huang and Figueiredo-Pereira, 2010). The nuclear inclusions in HD *in vivo* resemble nucleolar aggresomes *in vitro*, although they localize adjacent to the nucleoli and not similarly within the nucleolus (Davies et al., 1997). Upon treatment of sensory ganglion neurons with proteasome inhibitors, and in motor neurons with severe dysfunction of proteostasis in a mouse SMA model, nuclear poly(A) RNA granules are formed frequently adjacent to the nucleolus, but not within it (Palanca et al., 2014; Narcís et al., 2018). These data may indicate differences in the *in vivo* vs. *in vitro* nucleolar state, as in post-replicative cells the nucleoli have adhered to a fully mature form. On the other hand, SUMO1-positive intranucleolar spots lacking nascent RNA and associated with a nucleolar reorganization of fibrillar centers have been found *in vivo* in motor neurons in the spinal muscular atrophy (SMA) (Tapia et al., 2017). Furthermore, nucleolar aggresomes have been shown to occur in *ex vivo* prostate tissue (Latonen et al., 2011) and in human breast and prostate cancer tissue (Audas et al., 2016).

Although the focus on the nucleoli with respect to neurodegeneration has been on effects of diseased mutants on ribosomal production and activity, several neurodegeneration relevant proteins have been shown to localize to nucleoli. A specific form of mutant Htt localizes to the nucleolus in mouse neuronal progenitor cells (Trettel et al., 2000). In addition, artificial β-sheet proteins known to form prefibrillar and fibrillar aggregates have been shown to accumulate to nucleoli in cultured cells (Woerner et al., 2016). The most compelling *in vitro* evidence for nucleolar aggresome relevance for neurodegenerative disease exists for C9orf72. Expansion of the a GGGGCC hexanucleotide repeat in the first intron of this gene is the most common genetic alteration leading to hereditable amyotrophic lateral sclerosis (ALS). Glycine/arginine and proline/arginine repeats resulting from non-ATG translation of these repeats are recruited to nucleoli and hamper ribosome biogenesis, resulting in cell death (Kwon et al., 2014). Accumulating evidence shows that these dipeptide repeats locate to the GC where they phase-separate with NPM, disrupting nucleolar function (Haeusler et al., 2014; Lee et al., 2016; Lin et al., 2016). These repeats in fact interact with several IDR-containing proteins, many being RNA binding proteins (RBPs) and/or MLO proteins (Lee et al., 2016). While many repeat-expanded proteins accumulate to ribonucleoprotein (RNP) granules (Van Treeck and Parker, 2018), it is not clear what dictates accumulation of C9orf72 repeat peptides to nucleoli. Interestingly, the dipeptide repeats of C9orf72 have been shown to function as polyamines and promote intermolecular RNA-RNA interactions (Van Treeck et al., 2018). Although the functional significance of this in the toxicity of the mutant remains to be shown, it seems likely that these interactions affect the LLPS and/or nucleolar interactions of these peptides.

### Nucleolar Aggresomes, Cancer and p53

In general, the nucleolus and interactions with nucleolar proteins and rRNA species is central in regulation of certain tumor suppressor and oncogene activities, the most recognized being p53 and c-myc, respectively (Ruggero and Pandolfi, 2003; Boisvert et al., 2007). As nucleolar aggresomes have been detected in human breast and prostate cancer tissues (Audas et al., 2016), and they can be induced by proteasome inhibition in *ex vivo* prostate tissue (Latonen et al., 2011), nucleolar aggresomes may also be relevant for cancer. Dozens of cancer-related nuclear factors can be targeted to nucleolar aggresomes (Latonen, 2011; Latonen et al., 2011). While some of these have implications in regulation of nucleolar activity and ribosome production, such as c-Myc (reviewed in Lindström and Latonen, 2013), most have no identified function in the nucleoli and are likely regulatory targets of the aggresomes under stress.

p53 was one of the first proteins showed to exhibit stress-responsive nucleolar localization (Klibanov et al., 2001; Xirodimas et al., 2001; Latonen et al., 2003). p53 is a tumor suppressor and the most often mutated gene in human cancers (Muller and Vousden, 2013), and it has several connections to nucleolar-related proteins such as NPM, ARF and MDM2 (Mayer and Grummt, 2005). p53 and p53-derived fragments have been shown to aggregate *in vitro* (Silva et al., 2014), and several p53 mutants have been found as amyloid aggregates in tumor cell lines (Xu et al., 2011) and breast cancer biopsies (Levy et al., 2011). These aggregates inactivate p53 by sequestering the protein, thus blocking its transcriptional activity and pro-apoptotic function (Xu et al., 2011). A cell-penetrating peptide, ReACp53, designed to inhibit p53 amyloid formation, rescues p53 function in cancer cell lines and in organoids derived from high-grade serous ovarian carcinomas (HGSOC) (Soragni et al., 2016).

Tumor suppressor p53 translocates to nucleolus upon treatment of cells by proteasome inhibitors in cultured cells and *ex vivo* tissue (reviewed in Latonen, 2011). In addition, the chemical compounds PRIMA1 and PRIMA-1MET have been reported to induce nucleolar translocation of p53 (Rökaeus et al., 2007; Russo et al., 2010), although contradicting evidence also exists (Rangel et al., 2019). PRIMA1 is a mutant p53 reactivating compound (Bykov et al., 2002) which has been shown to reactivate unfolded p53 mutants to native, functional conformation and, recently, to prevent mutant p53 aggregate accumulation in cancer cells (Rangel et al., 2019). Thus, PRIMA-1 can rescue amyloid state of mutant p53, which has implications for future cancer treatment strategies (Rangel et al., 2019).

### Signals Behind Nucleolar Localization of Proteins

Signals in amino acid sequence that target proteins to the nucleolus are referred to as nucleolar localization signals (NoLS). They are arginine/lysine rich and range from seven to approximately 30 aa residues, but they are relatively rare and not a requirement for nucleolar localization. In fact, nucleolar localization of a protein is viewed to most often result from either direct or indirect interaction with nucleolar molecules, either rDNA, its transcripts, or protein components (reviewed in Emmott and Hiscox, 2009).

It is not clear what signals direct the localization and detention of extranucleolar proteins to nucleoli and nucleolar aggregates under stress. These likely depend on molecular interactions and involve changes in phase separation and transition balance due to presence of new molecules, but which specific molecules function in the seeding of the detention remain an open question for several conditions. Recently, using FUS family of proteins as an example it was shown that tyrosine residues in prion-like domains and arginine residues on RNA-binding domains govern the saturation concentration of phase separation (Wang J. et al., 2018). Interestingly, Mekhail et al. (2007) identified a common peptide motif in the proteins detained in the nucleoli during acidosis, including VHL, HSC70, RNF8 and cIAP2. This so called nucleolar detention signal regulated by H^+^ (NoDS^H+^) is different from the canonical nucleolar localization signal (NoLS) and is composed of an arginine motif combined to several hydrophobic repeats (Mekhail et al., 2007; Jacob et al., 2012). Up to 9% of all proteins harbor a NoDS, indicating that a substantial amount of the proteome may potentially be regulated in a similar fashion (Jacob et al., 2012). Thus, arginine rich motifs seem as a recurrent event in MLO and nucleolar targeting. The species of ncRNA expressed upon stress signals also have a role in recruiting the proteins to nucleolar aggresomes (discussed below), as specific RNAs, e.g., extended repetitive RNA motifs in clinical disorders, are capable of phase separation (Jain and Vale, 2017; Sawyer et al., 2018).

Post-translational modifications, especially ubiquitin family conjugates, may have a key role in localizing extranucleolar proteins to nucleolar aggresomes. In addition to ubiquitin and SUMO found in the physiological disease-relevant inclusion bodies (as discussed above), the nucleolar aggresomes have revealed several family members to be relevant for nucleolar aggresomes. The structures contain conjugated ubiquitin, indicating that at least some of the accumulated proteins harbor this modification (Latonen et al., 2011). Especially interesting is the role of SUMO-proteins, which are found in INBs and nucleolar aggresomes (Hutten et al., 2011; Latonen et al., 2011; Souquere et al., 2015). UBC9, the E2 SUMO-conjugating enzyme is also located in INBs, it’s depletion reduces INB size, and SUMO-1 mutant unable to conjugate proteins does not localize to INBs, indicating that SUMO conjugation is relevant for INB biology (Brun et al., 2017). Yet another ubiquitin homolog, NEDD8, was recently shown to localize to nucleolar aggresomes formed upon heat shock and proteasome inhibition (Maghames et al., 2018). Similarly to SUMO, this localization was linked to NEDD8 conjugation and even NEDD8/ubiquitin hybrid chain formation (Maghames et al., 2018). Thus it seems that nucleolar localization and aggregation of extranucleolar proteins is regulated by ubiquitin family of protein conjugation, requiring further investigation to understand the exact underlying mechanisms and functional consequences.

## Emerging Roles of Non-Coding Transcripts in the Nucleolus

The nucleolus is packed with non-coding RNA. After the 8S, 18S and 5.8S rRNAs have been transcribed by RNApolI and cleaved from their 47S precursor, they are post-transcriptionally modified through interaction with small nucleolar ribonucleoproteins (snoRNPs) and additional processing factors. For a long time, other rRNA sequences have been neglected as garbage sequences or non-specific degradation products. Recently, it has become clear that many non-coding RNA species in addition to the classical rRNA and snoRNA contribute to nucleolar biology. For example, rDNA is transcribed in antisense orientation to produce RNA contributing to epigenetic silencing of rDNA (Bierhoff et al., 2010).

Perhaps the most interesting resource of ncRNA in the rDNA sequence to be fully explored is the intergenic spacer (IGS). This sequence differs considerably from the rRNA coding sequences and has a high variability in nucleotide composition and length. Mayer et al. (2006) showed that some of these transcripts are required for establishing and maintaining a specific heterochomatic configuration at the promoter of a subset of rDNA arrays via NoRC, a chromatin remodeling complex. The transcripts here are 150–300 nt long and are complementary to the sequences in rDNA promoter (pRNA). During mid-S phase in the cell cycle, these pRNAs increase by 2-fold to repress rRNA synthesis in late replication (Santoro et al., 2010). Interestingly, the pRNA-dependent establishment of heterochromatin condensation of rRNA genes initiates highly condensed chromatin structures outside the nucleolus (Savic et al., 2014). This promotes transcriptional activation of differentiation genes, and is a mechanism shown to be inactivated in pluripotent embryonic stem cells (Savic et al., 2014). Thus, pRNA regulates chromatin plasticity and pluripotency.

Intergenic spacer also produces several stimuli-specific ncRNAs. Stress conditions such as heat shock and acidosis induce transcription of IGS to produce several transcripts shown to be involved in nucleolar aggresome formation, including IGS_16_RNA, IGS_22_RNA and IGS_28_RNA (Audas et al., 2012b; Jacob et al., 2012, 2013). These transcripts are produced from stimuli-specific loci (Audas et al., 2012a). Whether there are more IGS transcripts that are relevant for protein detention in the nucleolus and nucleolar aggresome formation remains to be investigated.

Although most lncRNAs are processed like typical mRNAs to be 5′ capped and 3′ polyadenylated, other lncRNAs are stabilized by alternative mechanisms. One mechanism for this adaptation of snoRNA processing to produce snoRNA-ended lncRNAs (sno-lncRNAs) and 5′ snoRNA-ended and 3′-polyadenylated lncRNAs (SPAs). Some sno-lncRNAs and SPAs have been shown to be involved in the regulation of pre-rRNA transcription and alternative splicing of pre-mRNAs (Xing and Chen, 2018). For example, a box H/ACA small nucleolar RNA (snoRNA)-ended long non-coding RNA (lncRNA) was described to enhance pre-rRNA transcription (SLERT) (Xing et al., 2017). SLERT requires box H/ACA snoRNAs at both ends for its biogenesis and translocation to the nucleolus. SLERT interacts with DEAD-box RNA helicase DDX21 via a 143-nt non-snoRNA sequence, following which DDX21 forms ring-shaped structures surrounding multiple RNApolI complexes and suppresses pre-rRNA transcription (Xing et al., 2017).

In *C. elegans*, there was recently a new class of antisense ribosomal siRNAs (risiRNAs) identified that downregulate pre-rRNA through the nuclear RNAi pathway (Zhou et al., 2017). risiRNAs exhibit sequence characteristics similar to 22G RNA while being complementary to 18S and 26S rRNA. risiRNAs induce translocation of the nuclear Argonaute protein NRDE-3 from the cytoplasm to nucleus and nucleolus, in which the risiRNA/NRDE complex binds to pre-rRNA and silences rRNA expression. Interestingly, exposing *Caenorhabditis elegans* to cold shock or UV radiation, risiRNAs accumulate, turning on the nuclear RNAi-mediated gene silencing pathway (Zhou et al., 2017). Whether similar mechanisms exist in mammalian cells and contribute to nucleolar stress responses remains to be explored.

## Concluding Remarks

It has become clear that, likely due to its phase separating propensities, the nucleolus can serve as a protective site for proteins following several environmental stimuli and stress signals. This detention may lead to formation of nucleolar aggresomes, and targets varying species of RNA and differential pools of proteins dependently on the cellular context and stress signal. What remains to be determined is the general mechanisms that dictates these responses. The requirements for the phase transition steps are to be studied in detail, and surely more RNA effectors are to be found. Investigation of protein amino acid sequence signals, regulation by conjugation of ubiquitin protein family members, and interactions between RNA-protein and protein-protein domains promoting aggregation and amyloid formation in the nucleolus may enlighten the cellular and molecular routes to target in pathological nuclear aggregation.

## Author Contributions

The author confirms being the sole contributor of this work and has approved it for publication.

## Conflict of Interest Statement

The author declares that the research was conducted in the absence of any commercial or financial relationships that could be construed as a potential conflict of interest.
